# H_2_O_2_-Induced Oxidative Stress Responses in *Eriocheir sinensis*: Antioxidant Defense and Immune Gene Expression Dynamics

**DOI:** 10.3390/antiox13050524

**Published:** 2024-04-26

**Authors:** Qinghong He, Wenrong Feng, Xue Chen, Yuanfeng Xu, Jun Zhou, Jianlin Li, Pao Xu, Yongkai Tang

**Affiliations:** 1 College of Fisheries and Life, Shanghai Ocean University, Shanghai 201306, China; hqh13990750864@163.com; 2 Key Laboratory of Freshwater Fisheries and Germplasm Resources Utilization, Ministry of Agriculture and Rural Affairs, Freshwater Fisheries Research Center, Chinese Academy of Fishery Sciences, Wuxi 214081, China; fengwenrong@ffrc.cn (W.F.); chenxue@ffrc.cn (X.C.); xuyuanfeng@ffrc.cn (Y.X.); lijl@ffrc.cn (J.L.); xup@ffrc.cn (P.X.); 3 Freshwater Fisheries Research Institute of Jiangsu Province, Nanjing 210017, China; finedrizzle@163.com

**Keywords:** *Eriocheir sinensis*, oxidative stress, antioxidation, gene expression, H_2_O_2_

## Abstract

*Eriocheir sinensis*, a key species in China’s freshwater aquaculture, is threatened by various diseases, which were verified to be closely associated with oxidative stress. This study aimed to investigate the response of *E. sinensis* to hydrogen peroxide (H_2_O_2_)-induced oxidative stress to understand the biological processes behind these diseases. Crabs were exposed to different concentrations of H_2_O_2_ and their antioxidant enzyme activities and gene expressions for defense and immunity were measured. Results showed that activities of antioxidant enzymes—specificallysuperoxide dismutase (SOD), catalase (CAT), total antioxidant capacity(T-AOC), glutathione (GSH), and glutathione peroxidase (GSH-Px)—varied with exposure concentration and duration, initially increasing then decreasing. Notably, SOD, GSH-Px, and T-AOC activities dropped below control levels at 96 h. Concurrently, oxidative damage markers, including malondialdehyde (MDA), H_2_O_2_, and 8-hydroxy-2′-deoxyguanosine (8-OHdG) levels, increased with exposure duration. The mRNA expression of *SOD*, *CAT*, and *GSH-Px* also showed an initial increase followed by a decrease, peaking at 72 h. The upregulation of *phenoloxidaseloxidase (proPO)* and *peroxinectin* (*PX*) was also detected, but *proPO* was suppressed under high levels of H_2_O_2_. *Heat shock protein 70* (*HSP70)* expression gradually increased with higher H_2_O_2_ concentrations, whereas *induced nitrogen monoxide synthase (iNOS)* was upregulated but decreased at 96 h. These findings emphasize H_2_O_2_’s significant impact on the crab’s oxidative and immune responses, highlighting the importance of understanding cellular stress responses for disease prevention and therapy development.

## 1. Introduction

*Eriocheir sinensis* holds the second highest rank in terms of production volume in the field of crustacean aquaculture in China. It is highly prized for its culinary attributes and economic value. *E. sinensis* has a long history of consumption in China. It has high content of protein, fats, and various vitamins [1]. The fatty paste and roe, that is, the gonads, represent luxury foodstuffs and are often featured in traditional Chinese cuisine. Over the years, there have been considerable advancements in the aquaculture techniques for *E. sinensis*. However, with an expansion in farm size and increased stocking densities, there has been a concomitant increase in the incidence of disease. This rise is attributable to combinations of environmental stressors and escalated pathogen load. The hepatopancreas of *E. sinensis* is not only an edible tissue but also serves multiple physiological functions. It is involved in digestion, absorption, and storage of nutrients, particularly during molting [2] and gonadal maturation [3]. Additionally, it plays a role in detoxification and metabolic regulation. Due to its important roles, the hepatopancreas is sensitive to changes in the external and internal environments. When *E. sinensis* is subjected to environmental stressors, its hepatopancreas is typically one of the first organs to be affected.

In aquaculture, *E. sinensis* is exposed to a variety of environmental stressors, such as hypoxia [4], elevated temperature [5], heavy metal ions [6], pesticides [7], and high levels of ammonia nitrogen [8]. These stressors trigger two types of stress responses: oxidative stress and nitrosative stress [9]. Oxidative stress induces the overproduction of reactive oxygen species (ROS), including superoxide anion radicals (O^2−^), hydroxyl radicals (OH), and H_2_O_2_. Nitrosative stress induces the production and release of nitric oxide (NO), triggering a series of chain reactions that result in the formation of reactive nitrogen species (RNS). Research has demonstrated that many diseases in *E. sinensis* are frequently accompanied by strong oxidative stress. Infections caused by *Aeromonas hydrophora* lead to an upregulation of antioxidant defenses, including T-AOC), GSH, and GSH-PX, as well as lysozyme (LZM) and phenoloxidase (PO) activities [10]. When *E. sinensis* was subjected to acute salt stress, there was an elevation in antioxidant enzyme activities, such as CAT, SOD, T-AOC, GSH-PX, and MDA levels, alongside an upregulation of the heat shock protein 90 (*HSP90*) gene, which enhanced resistance [11]. Conversely, under ammonia nitrogen stress, there was a notable decrease in the antioxidant capacity indicators of T-AOC, T-SOD, and GSH-Px in the hemolymph, alongside a significant increase in MDA, marking reduced antioxidant capacity and increased oxidative damage of *E. sinensis* [12]. Saline-alkali stress exposure resulted in initial increases followed by decreases in SOD, CAT, and T-AOC activities in the hepatopancreas of *E. sinensis*; the decreases in antioxidant capacity were in correlation with hepatopancreatic damage [13]. Yang’s work demonstrated that acute hepatopancreatic necrosis syndrome (AHPNS) in *E. sinensis* led to higher blood levels of aspartate aminotransferase (AST) and glutamic pyruvic transaminase (GPT) compared to healthy specimens; contrastingly, alkaline phosphatase (ALP) and acid phosphatase (ACP) activities in the hepatopancreas were notably lower, with a concurrent significant increase in MDA levels, indicating both oxidative stress and organ damage [14]. Thus, investigating the effects of oxidative stress on *E. sinensis* may offer new insights into exploring the pathogenesis of disease.

H_2_O_2_ is a widely prevalent ROS with a remarkable ability to penetrate cell membranes, leading to oxidative stress or triggering apoptosis within the cell. Consequently, H_2_O_2_ is frequently used as a standard reagent to experimentally induce oxidative stress in animals. In this study, one-year old juvenile *E. sinensis* were subjected to H_2_O_2_ stress tests to explore their physiological response to oxidative stress. The activity of antioxidase in the hepatopancreas and hemolymph, and the mRNA expression levels of antioxidative and immune-related genes in the hepatopancreas, were measured after 96 h of gradient H_2_O_2_ treatment. This research provides a scientific basis for the in-depth study of the oxidative stress response in *E. sinensis* and brings a novel perspective to the prevention and treatment strategies for diseases caused by oxidative damage.

## 2. Materials and Methods

### 2.1. Ethics Statement

The crabs were handled and the experimental procedures were performed in accordance with the guidelines for the care and use of animals for scientific purposes set by the Animal Ethics Committee of the Freshwater Fisheries Research Center (FFRC) Chinese Academy of Fishery Sciences, and the necessary ethical protocol code is LAECFFRC-2023-09-12. All operations were performed to minimize the suffering of the crabs.

### 2.2. Crabs and Rearing Conditions

Juvenile *E. sinensis* were obtained from Yangcheng Lake Shrimp and Crab Green Cultivation Base, Freshwater Fisheries Center, Chinese Academy of Fisheries Sciences. Juvenile crabs (13.34 ± 2.56 g) were acclimated to the aquatic environment in a laboratory aquarium (100 cm × 45 cm × 50 cm) for one week. During the acclimation period, continuous aeration was provided to maintain a dissolved oxygen concentration (DO) of ≥7.0 mg·L^−1^. The ambient water temperature was regulated at 25 ± 2 °C with a pH of 8.0 ± 0.2. Commercial feed was administered every morning, and one-third of the water volume was replaced every other day. Feeding was ceased 24 h prior to experimentation, and individuals in intermolt with healthy, intact appendages were selected for the study.

### 2.3. H_2_O_2_ Stress Treatment

Six treatment groups were set up in the experiment, with H_2_O_2_ concentrations set at 0 (control group), 3, 6, 9, 12, and 15 mmol·L^−1^. Each group containing 70 juvenile *E. sinensis* was raised separately in two tanks (100 cm × 45 cm × 50 cm) with the same conditions. During the experiment, the water was completely changed every 24 h, with the concentration of hydrogen peroxide being adjusted to meet the specified experimental requirements. During the experiment, the water quality parameters were maintained at a temperature of 25 ± 2 °C, DO ≥ 7.0 mg·L^−1^, pH = 8.0 ± 0.2, ammonia ≤ 0.02 mg·L^−1^, and nitrite ≤ 0.05 mg·L^−1^. Samples were collected at 0, 24, 48, 72, and 96 h of exposure. For each sample point, nine juvenile crabs were picked randomly and immediately anesthetized in an ice water bath. Hemolymph was extracted using a disposable sterile syringe from the basal membrane of the third walking leg, followed by dissection on ice for hepatopancreas sampling. After the hemolymph clotting at room temperature, it was centrifuged at 1000× *g* for 10 min to obtain serum. Hepatopancreas samples were flash-frozen in liquid nitrogen. Samples were stored at −80 °C for subsequent experiments.

### 2.4. Biochemical Analysis

Hepatopancreas tissues were immersed in physiological saline (with a weight/volume ratio of 1:9) and homogenized using a high throughput tissue grinder (SCIENTZ-48, Ningbo, China). After centrifuging at 5000× *g* for 15 min at 4 °C, the supernatant was collected for measurement. Serum was diluted using saline for enzyme activity determination. All parameters were determined using commercial assay kits according to the manufacturer’s protocols provided by Nanjing Jiancheng Bioengineering Institute (Nanjing, China). Total protein content (TP) was determined by the Coomassie Brilliant Blue assay (A045-2). MDA levels were assessed using the thiobarbituric acid (TBA) method (A003-1). T-AOC was measured via the ABTS method (A015-2-1). SOD activity was quantified through the nitro blue tetrazolium (NBT) method (A001-1), while CAT activity was evaluated using the ammonium molybdate method (A007-1-1). GSH (A006-2-1) and GSH-Px (A005-1) activities and H_2_O_2_ (A064-1-1) content were determined by colorimetric assay. The concentration of 8-OHdG was measured using a Crab 8-hydroxydeoxyguanosine Elisa Kit (H165-1).

### 2.5. Quantitative Real-Time Fluorescent PCR (qPCR) Analysis

Total RNA was extracted from the hepatopancreas by the TRIzol method. RNA quality, including purity and concentration, was assessed by spectrophotometry (NanoPhotometer^®^ N50, Implen, Munich, Germen) at 260/280 nm. The cDNA was synthesized from 2 μg of total RNA using the PrimeScript™ RT reagent kit with gDNA Eraser (Takara). Primers for *SOD*, *CAT*, *GPS-Px*, *iNOS*, *HSP70*, *PX*, *proPO*, and *β-actin* were designed by the Primer Premier 5.0 software (USA) based on known sequences from *E. sinensis*. The primer sequences and GenBank accession numbers are listed in Table 1. *β-actin* served as the internal reference gene. The qPCR was performed on a Thermal Cycler Dice^®^ Real Time System TP800 and programmed as follows: an initial denaturation step at 95 °C for 30 s, followed by 40 cycles of denaturation at 95 °C for 5 s and annealing at 60 °C for 30 s. The melting curve analysis was performed with the following temperatures and times: 95 °C for 15 s, 60 °C for 30 s, 95 °C for 5 s. Three replicates were performed for each sample. Each sample was subjected to three repetitions, and the data were converted to cycle/threshold (Ct) values after each reaction. The relative gene expression levels were calculated by the 2^−ΔΔCt^ method.

### 2.6. Data Analysis

The results are expressed as mean ± standard error (mean ± SE). Data analysis was conducted using SPSS Statistic 23.0 software (IBM, Armonk, NY, USA), with one-way analysis of variance (ANOVA) employed to evaluate differences among groups, and the Tukey test was used for post hoc comparisons to assess the significance of differences between groups (*p* < 0.05). Tests for homogeneity of variance were utilized to verify the assumption of normal distribution of the data. Graphical representations were generated using GraphPad Prism 8.0.

## 3. Results

### 3.1. Effect of H_2_O_2_ Stress on Antioxidant Response in Hepatopancreas

**Figure 1:**
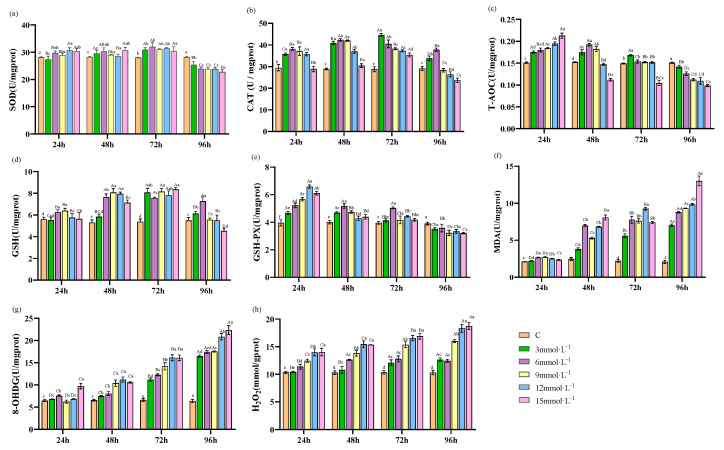
Effects of H_2_O_2_ stress on the antioxidant parameters of hepatopancreas. Distinct lowercase letters indicate significant differences at the same time point (*p* < 0.05), and distinct uppercase letters indicate significant differences at different time points within the same treatment group (*p* < 0.05).

Following exposure to H_2_O_2_ stress, the SOD activity in hepatopancreas showed an initially increasing and subsequently declining response over time. Notably, the activities peaked at 72 h and were significantly lower than in the control at 96 h of stress (*p* < 0.05, Figure 1a) in all the treated groups. The CAT activity displayed a similar trend, showed a rise and subsequent fall over the course of the experiment, reaching peak levels at 48 h for concentrations of 3 mmol·L^−1^ and 15 mmol·L^−1^, and at 72 h for concentrations of 6 mmol·L^−1^, 9 mmol·L^−1^, and 12 mmol·L^−1^. Notably, CAT activities at 12 mmol·L^−1^ and 15 mmol·L^−1^ significantly diminished compared to those of the control at 96 h (*p* < 0.05, Figure 1b). T-AOC within the 6 and 9 mmol·L^−1^ treatment groups showed an initial increase, peaking at 48 h, while the 12 and 15 mmol·L^−1^ groups peaked at 24 h before exhibiting a downward trend. At 96 h of stress, T-AOC levels in all treatment groups were significantly reduced compared to those of the control group (*p* < 0.05, Figure 1c). GSH levels in treated groups also rose and then fell, with the greatest levels observed at 72 h. Notably, the 15 mmol·L^−1^ treatment group showed a significantly lower GSH activity than the control at 96 h (*p* < 0.05, Figure 1d). GSH-PX activity in the treated groups displayed an initial rise followed by a decline, with activities substantially lower than those of the control group at 96 h of stress (*p* < 0.05, Figure 1e). MDA, 8-OHdG, and H_2_O_2_ levels all exhibited a consistent upward trend in response to both increased experimental duration and elevated stress concentrations (Figure 1f–h).

### 3.2. Effect of H_2_O_2_ Stress on Antioxidant Response in Hemolymph

**Figure 2:**
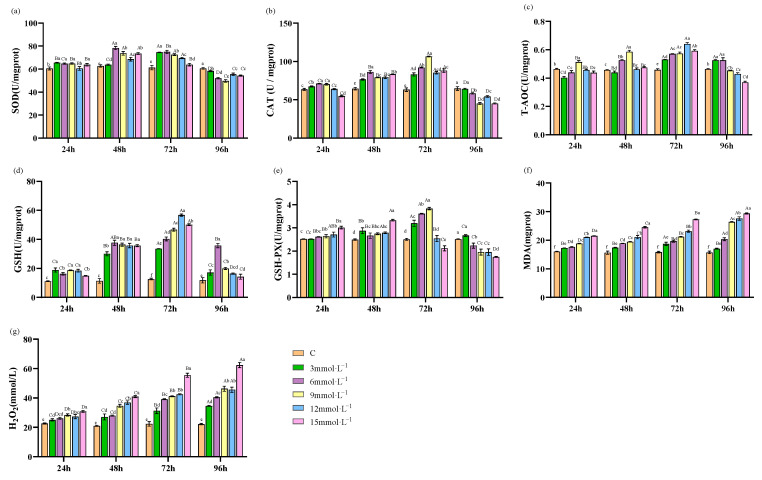
Effects of H_2_O_2_ stress on the antioxidant parameters of hemolymph. Distinct lowercase letters indicate significant differences at the same time point (*p* < 0.05), and distinct uppercase letters indicate significant differences at different time points within the same treatment group (*p* < 0.05).

The activities of SOD, T-AOC, CAT, GSH, and GSH-PX in all treatment groups initially increased and subsequently decreased over the duration of the experiment. Specifically, SOD activity in the 3 and 12 mmol·L^−1^ H_2_O_2_ treatment groups reached a maximum at 72 h, while peak activity in the other concentration groups occurred at 48 h (Figure 2a). T-AOC, CAT, and GSH activities reached their respective maxima at 72 h (Figure 2b–d). GSH-PX activity showed a peak at 48 h in the 12 and 15 mmol·L^−1^ H_2_O_2_ treatment groups, and at 72 h in the lower concentration groups of 3, 6, and 9 mmol·L^−1^ (Figure 2e). Conversely, the concentrations of MDA and H_2_O_2_ in the hemolymph showed an increasing trend with experimental time. The concentrations of MDA and H_2_O_2_ in the hemolymph exhibited a progressively increasing trend as time continued (Figure 2f,g).

### 3.3. Effect of H_2_O_2_ Stress on Genes Expression in Hepatopancreas

**Figure 3:**
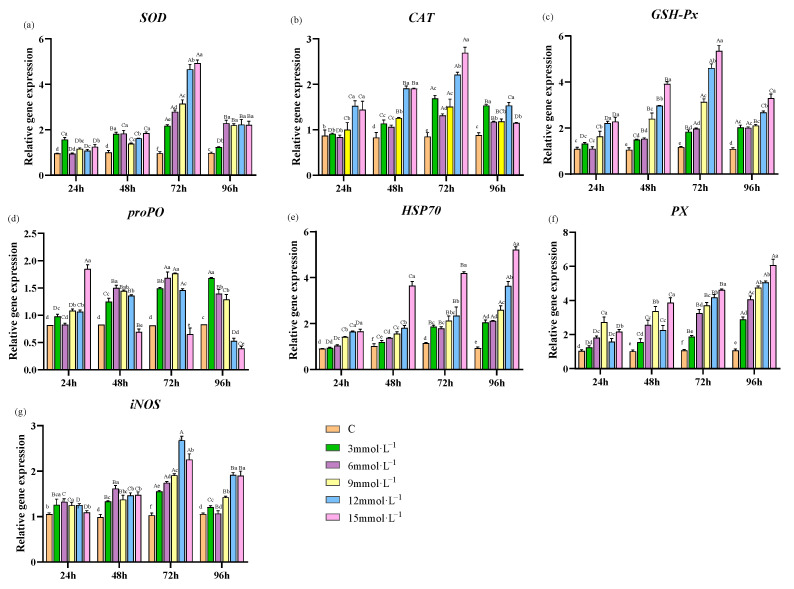
Effects of H_2_O_2_ stress on the expression of antioxidant-related genes in hepatopancreas. Distinct lowercase letters indicate significant differences at the same time point (*p* < 0.05), and distinct uppercase letters indicate significant differences at different time points within the same treatment group (*p* < 0.05).

During H_2_O_2_-induced stress, the mRNA expression levels of *SOD*, *CAT*, *GSH-Px*, and *iNOS* in each treatment group exhibited a tendency to increase and then decrease over time. These expression levels of genes peaked at 72 h post-treatment. By 96 h, the mRNA expression across all stressed groups was significantly increasing compared to the control (*p* < 0.05, Figure 3a–c,g). Concurrently, the mRNA expression of *proPO* in the 3, 6, 9, and 12 mmol·L^−1^ H_2_O_2_ concentrations of different treatment groups also increased and then decreased, reaching the highest value at 72 h. Notably, in the 15 mmol·L^−1^ H_2_O_2_ concentration group, the mRNA expression of *proPO* demonstrated a consistent decline over time and was significantly reduced compared to that of the control group at 48 h (*p* < 0.05, Figure 3d). Additionally, the mRNA expression levels of *HSP70* and *PX* showed a steady increase as the duration of time extended (Figure 3e,f).

## 4. Discussion

Extensive studies have demonstrated that variations in salinity, alkalinity, dissolved oxygen, temperature, and ammonia nitrogen within the aquatic environment can induce defense responses in organisms, including oxidative stress responses [15,16,17,18]. Under such stress conditions, the continuous production of ROS can disrupt the balance between the oxidative and antioxidant system, inflicting oxidative damage on lipids, proteins, DNA, and carbohydrates. When subjected to external stressors, the antioxidant system responds swiftly, enhancing its antioxidative capacity and modulating the expression of relevant genes to mitigate the stresses of oxidative challenge.

### 4.1. Effect of H_2_O_2_ Stress on Antioxidative Enzyme Activities in Hepatopancreas of E. sinensis

Under normal physiological conditions, organisms generate ROS as a byproduct of metabolism. However, an excessive accumulation of ROS can negatively impact the organism’s physiological state [19]. Antioxidants, which organisms intrinsically possess, can promptly and effectively remove ROS, thereby preventing oxidative stress. Hepatopancreas of *E. sinensis* plays a key role in eliminating excessive ROS [20]. Under stress conditions, SOD and CAT are critical antioxidant enzymes and function as the primary line of defense against the overproduction of ROS, mitigating potential adverse effects [21]. SOD removes the conversion of superoxide radicals (·O^2−^) into H_2_O_2_ and O_2_, while CAT further decomposes H_2_O_2_ into water (H_2_O) and oxygen (O_2_). Their combined activity effectively eliminates oxidative damage from superoxide radicals, thus preserving the organism’s internal homeostasis [22]. T-AOC is the cumulative antioxidant potential of tissue and represents the organism’s overall capacity to scavenge ROS [23]. Studies showed that air exposure caused oxidative stress in *E. sinensis*, with the activities of SOD and CAT in the hepatopancreas initially increasing and then decreasing as the duration of exposure extended [24]. In the Pacific white shrimp (*Litopenaeus vannamei*), SOD and CAT activities in the hepatopancreas were found to be elevated by hypoxia treatment; however, post-reoxygenation, the activities first rose and then diminished [25]. Furthermore, after recovery from cold-shock treatment in Pacific white shrimp, activities of SOD, CAT, and T-AOC showed an initial increase followed by a gradual decrease [26]. The results of our study indicated that during H_2_O_2_ stress, SOD, CAT, and T-AOC levels in the hepatopancreas initially increased and then decreased. This suggests that the antioxidative capacity of the organism was rapidly enhanced in response to H_2_O_2_ stress. Firstly, the presence of H_2_O_2_ directly enhanced CAT activity. Additionally, oxidative stress elevated·O^2−^ levels, which increased SOD activity. SOD converted·O^2−^ into H_2_O_2_, which in turn boosted CAT activity to eliminate excess H_2_O_2_. This resulted in an elevated activity of T-AOC. However, with the persistence of oxidative stress, the antioxidant system exceeded its reductive limit, leading to oxidative damage. This occurred when the cell failed to counterbalance the damage or the synthesis of new enzymes became impaired, as Sohal, R.S. indicated [27], which subsequently led to a decrease in antioxidant activities.

GSH possesses the capability to scavenge ROS, including free radicals, peroxides, and lipid peroxides, thereby playing a crucial role in cellular antioxidative defense mechanisms [28]. GSH-Px is an important peroxidolytic enzyme, catalyzing the specific reduction of ROS by oxidizing reduced GSH to its oxidized form against lipid peroxidation [29]. Wang et al. found that the administration of aflatoxin B1 to *L. vannamei* significantly increased the activities of CAT, SOD, and GSH-PX in the hepatopancreas compared to controls, with a tendency to increase and then decrease [30]. Duan et al. studied the oxidative stress response of *Penaeus monodon* to *Vibrio parahaemolyticus* infection, noting that GSH-Px and SOD activities in the hepatopancreas initially increased and then decreased, while the MDA content persistently rose [31]. In our study, when exposed to H_2_O_2_, the activities of GSH and GSH-Px both exhibited a trend of initial increase followed by a decrease. Specifically, the activity of GSH-PX peaked at 24 h post-stress and then progressively decreased from 48 h to 96 h. Meanwhile, GSH activity reached a higher level at 48 h and 72 h. This pattern may be due to the role of GSH as the substrate for GSH-Px. Increased activity of GSH-Px led to the consumption of GSH. Notably, the activity of GSH-Px decreased after 48 h, which consequently led to a continued rise in GSH levels [32]. At a H_2_O_2_ concentration of 15 mmol·L^−1^, GSH and GSH-Px activities showed a significant reduction compared to those of the control. This reduction may be attributed to exacerbated lipid peroxidation, resulting in hepatopancreatic damage compared to the control.

H_2_O_2_ is a significant byproduct of oxidative stress that belongs to ROS. MDA, a typical product of ROS-induced lipid peroxidation, serves as a crucial indicator of oxidative stress, reflects the rate and intensity of lipid peroxidation, and indirectly indicates the degree of tissue peroxidative damage [33,34]. When ROS attack DNA molecules, 8-OHdG is formed as an oxidative adduct. It is widely recognized as a sensitive biomarker for oxidative DNA damage [35]. Lin et al. found that when Cd stress was applied to *E. sinensis*, the activities of SOD, CAT, and GPx followed a trend of initial increase and then decrease, coinciding with increased MDA and H_2_O_2_ content, which led to tissue damage and apoptosis [36]. When *Charybdis japonica* was exposed to sulfide, MDA content had an ascending trend [37]. Additionally, the hepatopancreatic cells of *E. sinensis* showed an increase in 8-OHdG content after in vitro stimulation with abamectin, indicating DNA damage [38]. In red swamp crayfish (*Procambarus clarkii*), there was a significant increase in 8-OHdG levels in response to the pesticide deltamethrin [39]. The results of our experiment showed that under H_2_O_2_ stress, the MDA and H_2_O_2_ levels in the hepatopancreas of *E. sinensis* showed a gradual increase. This indicates that the production and accumulation of ROS in the hepatopancreas led to aggravative lipid peroxidation. Additionally, the significant elevation in the levels of 8-OHdG observed after 48 h of exposure highlights a time-dependent aggravation of oxidative DNA damage.

### 4.2. Effects of H_2_O_2_ Stress on the Antioxidant Enzyme Activities of Hemolymph in E. sinensis

Crustaceans depend on the innate immune defense system to combat infections. The hemolymph serves as the primary vehicle for immunological defense and is vital in mediating the host’s defensive reactions [40]. Singaram et al. observed that in the mud crab (*Scylla serrata*), antioxidant parameters such as SOD, CAT, and GPx in the hemolymph increased initially and then decreased when exposed to mercury stress [41]. Similarly, *E. sinensis* exhibited a comparable response under thiamethoxam stress, with activities of SOD, CAT, T-AOC, and GSH-Px in the hemolymph showing an initial rise followed by a decline [42]. Furthermore, when *E. sinensis* was subjected to acute ammonia-N stress, there was a significant decrease in hemolymph antioxidants T-AOC and T-SOD, while levels of GSH-Px and MDA were concomitantly elevated [12]. In our study, the levels of SOD, CAT, and T-AOC in the hemolymph showed an initial increase followed by a subsequent decrease under H_2_O_2_-induced oxidative stress. This indicates that the crab initially upregulated SOD and CAT activity to counteract the accumulation of ROS. However, as the duration of stress extended, the activities of these antioxidant enzymes became suppressed. This could be due to an excessive accumulation of ROS exceeding the detoxification capacity of SOD and CAT, and subsequently leading to a reduction in T-AOC activity. Moreover, GSH and GSH-Px activities showed a tendency of initial increase and then decrease. This pattern triggered by the initial accumulation of peroxides in hemolymph. But, theree activities decreased when oxidative stress was overwhelmed at a particular ROS threshold. Furthermore, upon exposure to deltamethrin, *E. sinensis* exhibited a significant elevation in oxidative stress markers H_2_O_2_ and MDA in the hemolymph [43]. Similarly, our findings revealed a consistent increase in MDA and H_2_O_2_ levels in the hemolymph of *E. sinensis* under H_2_O_2_ stress. The results indicated that H_2_O_2_, acting as inducer, can lead to significant accumulation in both ROS and lipid peroxidation products in the hemolymph.

### 4.3. Effects of H_2_O_2_ Stress on the Expression of Antioxidant- and Immune-Related Genes in Hepatopancreas

In *E. sinensis*, the innate immune system is principally composed of the antioxidant systems, prophenoloxidase (proPO) system, and multiple immune factors [44]. Among them, key antioxidant enzymes such as SOD, CAT, and GPx are the first line of defense against external invasions [45]. Studies have shown that the mRNA expression of *SOD*, *CAT*, and *GPx* in the hepatopancreas of *Portunus trituberculatus* initially increased and then decreased when infected by *Plasmodium trituberculatus* [46]. Similarly, the kuruma prawn (*Marsupenaeus japonicas*) exhibited an initial increase and subsequent decrease in these mRNA expressions when subjected to nitrite stress [47]. In *L. vannamei*, a comparable trend in the mRNA expression of *CAT* and *GPx* was observed after recovery from cold shock [26]. In this study, the expression of *SOD*, *CAT*, and *GSH-Px* first increased and then decreased under H_2_O_2_ stress, reaching the peak at 72 h, consistent with the activity profiles of these enzymes. This indicates that oxidative stress triggered the gene expression of antioxidant enzymes in the hepatopancreas. However, as the stress intensified, the levels of gene expression decreased.

The proPO system, significantly implicated in the melanization process, is integral to crustacean immune responses and participates in the acute reaction to pathogenic challenges [48]. It is a complex cascade consisting of proPO, PO, pattern recognition proteins (PRPs), and multiple serine proteases. Upon invasion by external pathogens, PRPs initiate a cascade of reactions that activates proPO into its active form, PO. Within this system, an important immune factor known as PX is also activated alongside proPO, thereby acquiring biological activity [49]. Studies have revealed distinct responses of genes in crustaceans’ proPO system following various challenges. In *P. clarkii*, infection with *Aeromonas veronii* led to an initial upregulation followed by a downregulation of *proPO* expression [50]. Similarly, infection with *Aeromonas astaci* in Japanese water shrimp (*Macrobrachium nipponense*) caused a significant upregulation of *proPO* mRNA levels [51]. Additionally, the immunostimulant β-glucan was found to induce an upregulation of *PX* in the Indian white shrimp (*Fenneropenaeus indicus*) [52]. In the current study, consistent elevation of *proPO* mRNA was observed at the H_2_O_2_ concentration of 3 mmol·L^−1^. Conversely, H_2_O_2_ concentrations of 6, 9, 12, and 15 mmol·L^−1^ induced an initial increase and then a decrease in *proPO* mRNA levels. This indicates that while low concentrations of H_2_O_2_ activated the proPO system, excessively high levels may disrupt it, causing a decrease in *proPO* mRNA expression. Moreover, the relative expression of *PX* continuously increased over time with rising H_2_O_2_ concentrations, suggesting that *PX* expression increased in accordance with oxidative stress, thereby enhancing the immune system and disease resistance of *E. sinensis*.

Heat shock proteins (HSPs) are ubiquitously distributed within the cells of both eukaryotes and prokaryotes with a highly conserved evolutionary process. HSPs perform multiple biomolecular functions, including as molecular chaperones, antioxidants, regulators of apoptotic, and mediators of immune responses [53]. As sensitive biomarkers of environmental stress, HSPs can provide indications to diverse stressors, such as water environmental factors, salinity, air exposure, and pesticides—all of which can elicit an increase in *HSP* expression levels [54,55,56,57]. When ridgetail white shrimp (*Exopalaemon carinicauda*) were exposed to *Prorocentrum minimum*, an increase in *HSP70* gene expression was observed in hemocytes and the hepatopancreas [54]. In *Macrophthalmus japonicus*, the mRNA expression of *HSP70* and *HSP90* was significantly upregulated in the hepatopancreas under salinity or bisphenol A (BPA) stress [55]. Under conditions of air exposure, mud crab (*Scylla paramamosain*) exhibited raised levels of *HSP90* and *HSP70* mRNAs in the hepatopancreas [56]. Moreover, in the black tiger prawn (*Penaeus maculatus*), *HSP70* in the muscle was significantly increased under the stress of the pesticides endosulfan and deltamethrin [57]. However, *E. sinensis* showed an initial increase followed by a decrease in *HSP70* gene expression when exposed to glyphosate [58]. In the current study, the expression of *HSP70* in the hepatopancreas of *E. sinensis* showed a continuous increase under H_2_O_2_ stress, correlating with both the duration of exposure and rising H_2_O_2_ concentrations. Notably, at a higher concentration of 15 mmol·L^−1^, a substantial upsurge in expression was observed. The study demonstrated that the elevated expression of *HSP70* may play a crucial role in mitigating oxidative damage.

*iNOS* is a vital component of the innate immune system, possessing antiviral, antibacterial, and antiparasitic properties. iNOS exerts these effects by directly or indirectly targeting the bases and chains of DNA, proteins, and membrane lipids, thereby inflicting damage to the DNA, enzymes, and membranes of pathogens [59]. Post-infection with the White Spot Syndrome Virus (WSSV), the expression of *iNOS* in Chinese white shrimp (*Fenneropenaeus chinensis*) and *M. japonicas* showed an initial upregulation followed by a subsequent reduction [60]. Similarly, *S. paramamosain* showed a significant increase at the mRNA levels of *NOS* within the intestine, hepatopancreas, and hemocytes upon pathogens infection, suggesting a correlation between *NOS* activity and immune system functionality [61]. In this study, the expression of iNOS in *E. sinensis* during H_2_O_2_ stress also followed a trend of first increasing and then decreasing, indicating the role of *iNOS* in modulating the immune response of *E. sinensis*. Additionally, iNOS has the ability to generate NO, which may lead to an increase in RNS, and thereby intensify damage in *E. sinensis* [62].

### 4.4. Effects of Stressors on the Antioxidant Capacity of Crustaceans

The crustacean antioxidant enzyme system plays a pivotal role in combating oxidative stress, representing an intricate mechanism by which these organisms maintain physiological homeostasis amidst environmental perturbations. We compared the trends in oxidative stress markers in crustaceans under various stressors to gain deeper insight into their physiological responses to oxidative stress over time (Table S1). We observed that different stress treatments elicited varying antioxidant responses across different crustaceans. For crustaceans, the primary external stressors include environmental pressures such as temperature, hypoxia, salinity, ammonia, and desiccation; and anthropogenic stressors such as heavy metals (copper, cadmium), toxic substances, and pesticides (aflatoxin, bisphenol, abamectin, deltamethrin). Furthermore, pathogenic microorganisms (bacteria and viruses), compound the oxidative burden. In response to oxidative stress, the activity of antioxidant enzymes in crustaceans can exhibit three distinct trends: an increase, a decrease, or an initial increase followed by a decrease. Generally, upon exposure to stressors, there is an upregulation of antioxidant markers to neutralize the surge in ROS. However, a decrease in certain antioxidant markers may occur due to depletion in response to excessive ROS or as a result of tissue damage. Additionally, antioxidant markers initially rise due to the pro-oxidant characteristics; if the stress is prolonged or excessive, the antioxidant system may become depleted or damaged, leading to a decrease in the activity of antioxidant enzymes. This inability to effectively clear ROS aggravates cellular damage. The elevation of lipid peroxidation products (such as MDA) and DNA damage markers (such as 8-OHdG) are also significant indicators of oxidative stress, signaling damage to cell membranes and genetic material.

## 5. Conclusions

In summary, we exposed *E. sinensis* to various concentrations of H_2_O_2_ and monitored physiological and biochemical markers of oxidative stress. We also measured expression levels of genes associated with the antioxidant response and immune function. The conveyed data support the dynamic and biphasic nature of the oxidative stress response in *E. sinensis*. The findings demonstrate that an organism’s initial response to oxidative stress is to enhance its antioxidative defenses. However, if the intensity or duration of the stress surpasses a certain threshold, the protective mechanisms become overwhelmed, resulting in oxidative damage. These findings have significant implications for comprehending the stress responses at the cellular and molecular levels and can be critical for devising strategies to shield organisms from oxidative harm.

## Figures and Tables

**Table 1:** Sequences of primers used in qPCR.

Gene	Primer Sequence (5′-3′)	Product Length (bp)	GenBank Accession Number
*iNOS*	TTGCCAGAGCCGTCAAGTT GCGCCTCGTGTTCTATGTTG	201	XM_050876720.1
*GSH-Px*	ATCCTGTACCCTGCAACCAC CTCTGGGAACAGCTTCTTGG	174	FJ617305.1
*SOD*	TGGACTGACGGAAGGGCTGC TGGCGTTAGGGGCGGAGTG	128	FJ617306.1
*CAT*	CCTGCTCGCAGGAATCGGTG GTCCAAGGAGGTGGCGGTCA	159	MH178391.1
*HSP70*	GGCAAGGCAGCGAAGGTCATC CGGCATTGGTGACAGACTGACG	127	KC493625.1
*peroxinectin*	CAGCAACGACTACAACCCGA TCCTTGCACCAGGGAATGAC	91	GU353176.1
*Prophenoloxidase*	CCATGTCATCATTGCAGCGG TGTACTTGTGCCAGCGGTAG	119	EF493829.1
*β-actin*	TGGGTATGGAATCCGTTGGC AGACAGAACGTTGTTGGCGA	101	KM244725.1

## Data Availability

The original contributions presented in the study are included in the article/Supplementary Materials; further inquiries can be directed to the corresponding authors.

## Supplementary Materials

The following supporting information can be downloaded at: https://www.mdpi.com/article/10.3390/antiox13050524/s1. References [63,64,65,66,67,68,69,70,71,72,73,74] are cited in the Supplementary Materials.
