# Supplementary material for: H2O2-Induced Oxidative Stress Responses in Eriocheir sinensis: Antioxidant Defense and Immune Gene Expression Dynamics
Source: Antioxidants (Basel). 2024 Apr 26;13(5):524. doi: 10.3390/antiox13050524 (PMC11117496; doi:10.3390/antiox13050524)
Supplement: Supplementary file 1 [file antioxidants-13-00524-s001.zip › antioxidants-2925137-supplementary.pdf]

Table s1. Effects of stressors on the antioxidant capacity of decapoda crustaceans. ↓: decrease activity; ↑: increase activity.

species	Treatment	Changes in antioxidant enzyme activity	Duration	Growth stage	Tissue	Reference
Eriocheir sinensis	Hypoxia	SOD↑ CAT↑ T-AOC↑ MDA↑	24h	Juvenile	Gill, Muscles and hepatopancreas	[1]
	salinity	SOD↑CAT↑ T-AOC↑ GSH-Px↑ MDA↑	72h	Female adult	hepatopancreas	[2]
	cadmium	SOD↑↓ CAT↑↓ GSH-Px↑↓ MDA↑ H2O2↑	6d	adult	hepatopancreas	[3]
	ammonia	SOD↑↓ CAT↑↓ GSH-Px↓	24h	Juvenile	hepatopancreas	[4]
	saline-alkali	SOD↑↓ CAT↑↓ T-AOC↑↓ MDA↑	96h	Juvenile	hepatopancreas	[5]
	abamectin	MDA↑8-OHDG↑	24h	adult	hepatopancreas	[6]
Litopenaeus Vannamei	hypoxia	SOD↑↓ CAT↑↓ GSH-Px↑↓ MDA↑↓	24h	Juvenile	gill, hepatopancreas and hemolymph	[7]
	low temperature and air exposure	SOD↑↓CAT↑↓GSH-Px↑↓TAOC↑↓ MDA↑↓	24h	Juvenile	hepatopancreas and hemolymph	[8]
	Vibrio parahaemolyticus	SOD↓ GSH-Px↓ MDA↑	72h	Juvenile	serum	[9]
	aflatoxin B1	SOD↑↓CAT↑↓GSH-Px↑↓ MDA↑	30 d	Juvenile	Intestines and hepatopancreas	[10]
Penaeus monodon	*Vibrio parahaemolyticus*	SOD↑↓ CAT↓GSH-Px↑↓MDA↑↓	24h	Juvenile	Gill and hepatopancreas	[11]
	desiccation	SOD↑↓ CAT↓GSH-Px↑↓MDA↑	3h	Juvenile	hepatopancreas	[12]
Procambarus clarkii	acute hypoxia	SOD↑↓CAT↑↓GSH-Px↑↓	13h	adult	hepatopancreas	[13]
	polystyrene microplastics (PS)	SOD↑CAT↑GSH-Px↑↓ MDA↑	48h	Juvenile	hepatopancreas	[14]

	coppe (Cu)	SOD↑CAT↑GSH-Px↑↓ MDA↑↓				
	PS and Cu	SOD↑CAT↑↓GSH-Px↑↓ MDA↑↓				
	thermal stress	SOD↑↓CAT↑↓ T-AOC↑↓ MDA↑↓	96h	Juvenile	hepatopancreas	[15]
	silicone	SOD↑CAT↑ MDA↑	72h	adult	gill, muscle and carapace	[16]
	deltamethrin	8-OHDG↑	96h	Juvenile	serum	[17]
Charybdis japonica	sulfide	SOD↑↓CAT↑↓	96h	adult	hemolymph	[18]
	bisphenol A	SOD↑↓CAT↑↓ GSH-Px↑↓ MDA↑	15d	adult	hepatopancreas and hemolymph	[19]
Portunus trituberculatus	cold acclimation	SOD↑CAT↑ MDA↑	21d	adult	muscle and hepatopancreas	[20]
	cadmium	SOD↑↓CAT↑↓ GSH↑↓	96h	juvenile		[21]
Fenneropenaeus chinensis	cadmium	SOD↑↓CAT↓ GSH↑↓	96h	juvenile		[21]
	white spot syndrome virus (wssv)	SOD↑↓CAT↑↓T-AOC↑↓	72h	juvenile	gill, muscles,plasma, stomach and hepatopancreas	[22]

References:

[1]. Chen, X., et al., Alteration of antioxidant status, glucose metabolism, and hypoxia signal pathway in Eirocheir sinensis after acute hypoxic stress and reoxygenation. Comparative Biochemistry and Physiology Part C: Toxicology & Pharmacology, 2023. 268: p. 109604.

[2]. Wang, X., et al., Effects of acute salinity stress on osmoregulation, antioxidant capacity and physiological metabolism of female Chinese mitten crabs (Eriocheir sinensis). Aquaculture, 2022. 552: p. 737989.

[3]. Lin, Y., et al., Cell damage and apoptosis in the hepatopancreas of Eriocheir sinensis induced by cadmium. Aquatic toxicology, 2017. 190: p. 190-198.

[4]. Wang, T., et al., Metabolic changes and stress damage induced by ammonia exposure in juvenile Eriocheir sinensis. Ecotoxicology and Environmental Safety, 2021. 223: p. 112608.

[5]. Zhang, R., et al., Effects of saline-alkali stress on the tissue structure, antioxidation, immunocompetence and metabolomics of Eriocheir sinensis. The Science of the total environment, 2023. 871: p. 162109-162109.

[6]. Hong, Y., et al., Cytotoxicity induced by abamectin in hepatopancreas cells of Chinese mitten crab, Eriocheir sinensis: An in vitro assay. Ecotoxicology and Environmental Safety, 2023. 262: p. 115198.

[7]. Li, Y., et al., Oxidative stress, DNA damage and antioxidant enzyme activities in the pacific white shrimp (Litopenaeus vannamei) when exposed to hypoxia and reoxygenation. Chemosphere, 2016. 144: p. 234-240.

[8]. Xu, Z., et al., The oxidative stress and antioxidant responses of Litopenaeus vannamei to low temperature and air exposure. Fish & Shellfish Immunology, 2018. 72: p. 564-571.

[9]. Jiao, L., et al., Vibrio parahaemolyticus Infection Influenced Trace Element Homeostasis, Impaired Antioxidant Function, and Induced Inflammation Response in Litopenaeus vannamei. Biological Trace Element Research, 2021. 199(1): p. 329-337.

[10]. Wang, Y., et al., Aflatoxin B1 (AFB1) induced dysregulation of intestinal microbiota and damage of antioxidant system in pacific white shrimp (Litopenaeus vannamei). Aquaculture, 2018. 495: p. 940-947.

[11]. Duan, Y., et al., Oxidative stress response of the black tiger shrimp Penaeus monodon to Vibrio parahaemolyticus challenge. Fish & shellfish immunology, 2015. 46(2): p. 354-365.

[12]. Duan, Y., et al., Effect of desiccation on oxidative stress and antioxidant response of the black tiger shrimp Penaeus monodon. Fish & shellfish immunology, 2016. 58: p. 10-17.

[13]. Zhang, L., et al., Acute hypoxia and reoxygenation induces oxidative stress, glycometabolism, and oxygen transport change in red swamp crayfish (Procambarus clarkii): Application of transcriptome profiling in assessment of hypoxia. Aquaculture Reports, 2022. 23: p. 101029.

[14]. Zeng, Q., et al., Polystyrene microplastics enhanced copper-induced acute immunotoxicity in red swamp crayfish (Procambarus clarkii). Ecotoxicology and Environmental Safety, 2023. 249: p. 114432.

[15]. Ruan, G., et al., Short-term adaptability to non-hyperthermal stress: Antioxidant, immune and gut microbial responses in the red swamp crayfish, Procambarus clarkii. Aquaculture, 2022. 560: p. 738497.

[16]. Hossain, M.M., et al., Silicone stressed response of crayfish (Procambarus clarkii) in antioxidant

enzyme activity and related gene expression. Environmental Pollution, 2021. 274: p. 115836.
[17]. Hong, Y., et al., DNA damage, immunotoxicity, and neurotoxicity induced by deltamethrin on the freshwater crayfish,Procambarus clarkii. Environmental Toxicology, 2021. 36(1): p. 16-23.
[18]. Xu, X.H., et al., Immunological and histological responses to sulfide in the crab Charybdis japonica. Aquatic toxicology, 2014. 150: p. 144-150.
[19]. Peng, Y.Q., et al., Immunological responses in haemolymph and histologic changes in the hepatopancreas of Charybdis japonica (A. Milne-Edwards, 1861) (Decapoda: Brachyura: Portunidae) exposed to bisphenol A. Journal of Crustacean Biology, 2018. 38(4): p. 489-496.
[20]. Meng, X., et al., Physiological responses of swimming crab Portunus trituberculatus under cold acclimation: Antioxidant defense and heat shock proteins. Aquaculture, 2014. 434: p. 11-17.
[21]. Wang, S., et al., Toxicological responses of juvenile Chinese shrimp Fenneropenaeus chinensis and swimming crab Portunus trituberculatus exposed to cadmium. Ecotoxicology and Environmental Safety, 2022. 234: p. 113416.
[22]. Lu, Y., et al., Antioxidant responses of Fenneropenaeus chinensis to white spot syndrome virus challenge. Aquaculture international, 2020. 28(1): p. 139-151.
